# Ethnic Differences in the Incidence of Hypertension among Rural Chinese Adults: Results from Liaoning Province

**DOI:** 10.1371/journal.pone.0086867

**Published:** 2014-01-29

**Authors:** Zhaoqing Sun, Liqiang Zheng, Xingang Zhang, Jue Li, Dayi Hu, Yingxian Sun

**Affiliations:** 1 Division of Cardiology, Shengjing Hospital of China Medical University, Shenyang, P.R. China; 2 Heart, Lung and Blood Vessel Center, Tongji University, Shanghai, P.R. China; Shanghai Institute of Hypertension, China

## Abstract

**Background:**

This study was conducted to examine the differences in the incidence of hypertension and associated risk factors between Mongolian and Han populations in northeast China.

**Methods:**

A population-based sample of 4753 Mongolian subjects and 20,247 Han subjects aged ≥35 years and free from hypertension at baseline were followed from 2004–2006 to 2010. Incident hypertension was defined as systolic blood pressure≥140 mmHg, diastolic blood pressure ≥90 mmHg, or current use of antihypertensive medication.

**Results:**

During mean 4.3 years follow-up, a total of 8779 individuals developed hypertension. The age-adjusted incidence of hypertension for Mongolian subjects was 12.64 per 100 person-years, for Han subjects was 9.77 per 100 person-years (P<0.05). The incidence of hypertension was positively correlated with age, physical activity, drinking, body mass index (BMI), family of hypertension and prehypertension in the Han population. In the Mongolian population, hypertension was positively correlated with age, physical activity, education level, drinking, BMI, prehypertension and family history of hypertension. The rates of awareness, treatment and control of hypertension for newly developed cases among both Han and Mongolian populations were low. (36.5% vs. 42.3%, 13.1% vs. 18.2%, 0.7% vs. 1.3%, P<0.05, respectively).

**Conclusions:**

The incidence rate of hypertension is higher in the Mongolian populations than that in the Han populations, and hypertension in both ethnic populations was associated with similar risk factors. Our results suggest that most newly-diagnosed cases of hypertension are not adequately treated. Improvements in hypertension prevention and control programs in rural China are urgently needed.

## Introduction

Cardiovascular disease, including both stroke and heart disease, is now the leading cause of death among Chinese adults [1]. Hypertension is an important modifiable risk factor for cardiovascular disease and total mortality in the Chinese population [1]. Results of studies conducted in the 1980s and 1990s suggested that the prevalence and incidence of hypertension in rural China were very low [2]. However, China and, more specifically, rural China, has undergone rapid social change during the past two decades. For example, rural residents now periodically commute to urban regions where they are likely to acquire urban and Western lifestyles and dietary habits. Such changes are occurring all over China but they are particularly pronounced in the northeast and western sections of the country, where agricultural production is low and growth of urban centers is accelerating. More recent data also suggest a rapid increase in the number of cases of hypertension in rural China [3], [4]. Although the exact causes and mechanisms of hypertension are not known, it was generally believed that both genetic factors and environmental factors [5] are involved in determining the prevalence and incidence of hypertension.

There are 56 nationalities in China. Han is the largestethnicity. Mongolian is one of the minority nationalities. There are 5 million Mongolian people in China. Little was known about the genetic background and epidemiological data of hypertension in this population. Therefore, we compared the incidence of hypertension, and its associated risk factors between Mongolian and Han populations in northeast China.

## Methods

### Study design and study population

The study complies with the Declaration of Helsinki. The China Medical University Research Ethics Committee reviewered and approved the research protocol and that written informed consent was obtained from all subjects or their guardians. Subjects who agreed to participate were explained the content of informed consent which included the purpose of the study, medical items, and confidentiality agreement of personal information. Between 2004 and 2006, a multistage random cluster sampling design was used to select a representative sample from a rural population of 45,925 individuals aged 35 years and older from Fuxin country of Liaoning Province in northeastern China [3], [6]. In 2010, participants who were selected to participate at baseline were invited by the investigators to return to the clinic for follow-up. 3,579 were not included in the follow-up study because study participants' contact information was unavailable. Overall, 42,346 participants at baseline were eligible to participate in the follow-up study. 39,845 individuals completed the follow-up. Individuals with hypertension or other ethnic people at baseline were excluded. Our final analysis included 20,247 Han people and 4753 Mongolian people without hypertension at baseline.

### Baseline measurement

All baseline data was collected between 2004 and 2006 by well-trained local doctors using home visits. Prior to beginning the study, doctors received training regarding the purpose of the study, and how to administer the questionnaire, the method of measurement, and the study procedures. After this training a test was conducted and only those who obtained a perfect score were selected as investigators. Additionally, the investigators received further training and support throughout the period of data collection. Reliability analyses were conducted on baseline blood presure data collected by the investigators, which revealed high levels of inter- and intra-observer agreement. The intraclass correlation coeffeicient value for intra- and inter-observer agreement were 0.648 and 0.678, respectively.

A standardized questionnaire was used to collect demographic information (age, sex, and ethnicity), and socioeconomic data. Information on alcohol consumption, smoking habits, physical activity and hypertension history, and medication use, was also collected. Drinking status was assessed by alcohol consumption, which was defined as the weekly consumption of beer, wine and hard liquor converted into grams of alcohol. Current drinking was defined as alcohol consumption (≥8 g/week) [7]. Current smoking was defined as people who smoked at least one cigarette every day and continued for at least one year. Body weight and height were measured with subjects wearing light clothing and no shoes. Body mass index (BMI) was calculated by the weight in kilograms divided by height in square meters. Education level included high school education and not high school education. We defined middle school and college as high school education; defined elementary school, junior high school and illiteracy as not high school education. Physical activity included occupational and leisure-time physical activity. A detailed description of the methods has been presented elsewhere [8]. Occupational and leisure-time physical activity were merged and regrouped into three categories: (i) low—subjects who reported light levels of both occupational and leisure-time physical activity; (ii) moderate—subjects who reported moderate or high levels of either occupational or leisure-time physical activity; and (iii) high—subjects who reported a moderate or high level of both occupational and leisure-time physical activity.

A trained and certified observer used the American Heart Association protocol to perform three blood pressure measurements for each participant. The participant rested in a seated position for 5 min prior the measurements. Participants were advised to avoid alcohol consumption, cigarette smoking, coffee/tea, and exercise for at least 30 min before the measurements. The research staff used a standardized electronic sphygmomanometer (HEM-741C; Omron, Tokyo, Japan) and one of four cuff sizes (pediatric, regular adult, large, or thigh), which was chosen based on the basis of arm circumference. In this study, the terminal digit preference was used as an indicator of quality control for BP measurements. The terminal digit preference for each village was analyzed every 3 months. If digit preference was a major and persistent problem, villages were sent a reminder of the study target of 10% for each of the terminal digits. In summary, the end-digit preferences were observed for BP measurements, being prominent for the digit “0” (18.4% for systolic and 17.7% for diastolic BP) followed by a preference for the digit “5” (14.7% for systolic and 15.1% for diastolic BP). We used Joint National Committee on High Blood Pressure -7 (JNC-7) criteria [9], to define prehypertension (systolic blood pressure between 120 and 139 mmHg or diastolic blood pressure between 80 and 89 mmHg), hypertension (systolic blood pressure ≥140 mmHg, or average diastolic blood pressure ≥90 mmHg, and/or self-report of current treatment for hypertension with antihypertensive medication [4].

### Follow-up data collection

The follow-up examinations were conducted between July 2010 and October 2010. Three blood pressure measurements were taken according to a standard protocol identical to that used for the baseline examination. Use of antihypertensive medications was assessed by a standard questionnaire. Incident hypertension was defined as a mean systolic BP ≥140 mmHg, and/or diastolic BP ≥90 mmHg, and/or use of antihypertensive medication during the 2 weeks prior to the follow-up examination. Hypertension awareness, treatment and control were defined as follows: awareness was defined as a self-report of a prior health professional's diagnosis; treatment was defined as the current use of a prescription medication for hypertension management; control was defined as a current average systolic blood pressure <140 mmHg and an average diastolic blood pressure<90 mmHg [4].

### Statistical analysis

Baseline characteristics were compared between ethnic groups via t-tests for continuous variables and chi-squared tests for categorical variables. The crude incidence rate of hypertension by gender and blood pressure level was computed using a simple frequency variable as the number of new hypertension patients over the follow-up period divided by the size of the study population at baseline. The incidence rate was denoted by case load/100 000 per year and age adjusted to the 2000 world Health Organization standard population. A 95% CI was determined by Poisson model. Cox proportional hazards models were used to evaluate the association between hypertension incidence and possible predictors, which included age, education status, physical activity, BMI, family history of hypertension, blood pressure status, and smoking and drinking habits. Univariate analysis was initially used to evaluate the association between hypertension incidence and possible predictors. Then, the significant variables (P<0.10) from the unvariate analysis were entered to the multivariate Cox proportional hazards model (Forward stepwise). All analyses were separately conducted based on sex and ethnicity. All analyses were performed with SPSS statistical software version 12.0. A P value less than 0.05 was accepted as indicating statistical significance.

## Results

The demographic characteristics, health-related behaviors and lifestyle factors for Mongolian and Han populations by sex are shown in **Table 1**. For both Mongolian and Han subjects, when compared to women, men were more likely to drink or smoke, have blood pressure readings consistent with prehypertension, and have a family history of hypertension. Men also had higher overall blood pressure readings than women.

**Table 1:** Baseline characteristics of study participants.

Variables	Han (n = 20247)			Mongolian (n = 4753)		
	Man (n = 10326)	Women (n = 9921)	P value	Man (n = 2298)	Women (n = 2455)	P value
Age (years)*	48.53±10.67	47.96±10.40	<0.001	47.99±10.32	47.89±10.50	0.732
High school education (%)	5.6	3.7	<0.001	9.1	8.8	0.758
Physical activity (%)			<0.001			<0.001
Low	20.9	27.6		14.8	23.2	
Moderate	46.6	46.6		44.4	44.8	
High	32.5	25.8		40.8	32	
Current smoker (%)	66.1	14.3	<0.001	69.7	13.4	<0.001
Current drinker (%)	53.7	4.3	<0.001	64.6	7.1	<0.001
Family history of hypertension (%)	10.7	7.8	<0.001	12.1	9.1	<0.001
Systolic BP(mmHg)*	122.68±10.15	119.20±11.34	<0.001	123.81±9.72	120.02±11.23	<0.001
Diastolic BP(mmHg)*	76.78±7.32	75.15±7.77	<0.001	77.13±7.15	75.71±7.69	<0.001
Prehypertension (%)	76.1	63	<0.001	81.4	68.9	<0.001
BMI(kg/m2)*	22.78±2.68	23.01±3.04	<0.001	23.24±2.64	23.47±3.05	0.005

* Mean ± SD; BP, blood pressure; BMI: body mass index.

The incidence of hypertension in Han populations and Mongolian populations are shown in **Table 2**. During mean 4.3 years follow-up, 8779 individuals developed hypertension. The age-adjusted incidence rate of hypertension was 9.77 per 100 person-years (95% CI, 9.30 to 10.25) in the Han population and 12.64 per 100 person-years (95% CI, 11.56 to 13.73) in the Mongolian population. Both for men and women, Mongolian populations had higher incidence than that in the HAN populations (P<0.05).Among participants who were classified as having normal BP at baseline, the age-adjusted incidence rate of hypertension was 8.28 per 100 person-years (95% CI, 7.45 to 9.12) in the Han population and 10.57 per 100 person-years (95% CI, 8.44 to 12.70) in the Mongolian population. The incidence rate of hypertension among subjects with prehypertension at baseline was 10.36 per 100 person-years (95% CI, 9.79 to 10.93) in the Han population and 13.24 per 100 person-years (95% CI, 12.01 to 14.53) in the Mongolian population.

**Table 2:** Incidence of hypertension in HAN and MON of rural Chinese.

Variables	Han (n = 20247)				Mongolian (n = 4753)			
	case (n)	person-years of follow-up	crude rate (per 100 person-years, 95%CI)	age-adjusted rate (per 100 person-years,95%CI)	case(n)	person-years of follow-up	crude rate (per 100 person-years, 95%CI)	age-adjusted rate (per 100 person-years,95%CI)
Sex								
man	3724	38170	9.76(9.46–10.05)	10.68(9.99–11.36)	1095	8617	12.71* (12.01–13.41)	13.80* (12.16–15.44)
women	2965	38306	7.74(7.47–8.01)	8.86(8.21–9.51)	995	9675	10.28* (9.68–10.89)	11.62* (10.18–13.06)
Blood pressure								
prehypertension	5020	53261	9.43(9.18–9.67)	10.36(9.79–10.93)	439	13551	12.18* (11.63–12.73)	13.24* (12.01–14.53)
normal	1669	23215	7.19(6.86–7.52)	8.28(7.45–9.12)	1651	4741	9.26* (8.43–10.08)	10.57* (8.44–12.70)
Total	6689	76476	8.75(8.55–8.95)	9.77(9.30–10.25)	2090	18292	11.43* (10.97–11.89)	12.64* (11.56–13.73)

95%CI: 95% confidence interval;

* *P*<0.05, compared with HAN populations.

**Figure 1:**
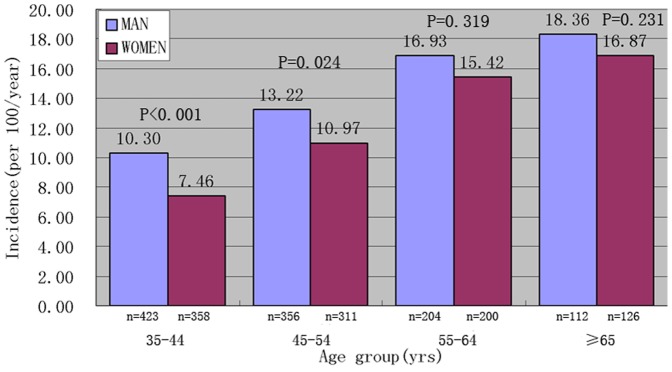
Incidence of hypertension in Mon by age group and sex.

**Figure 2:**
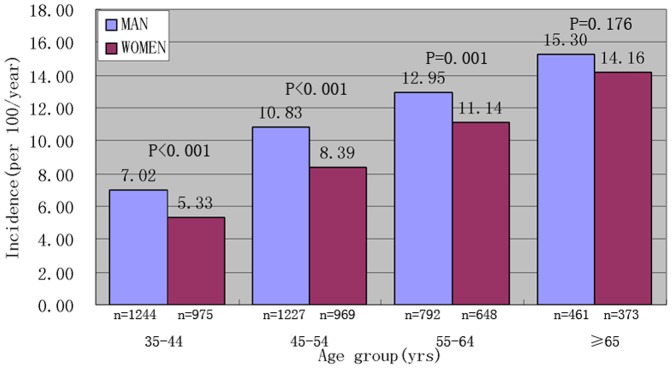
Incidence of hypertension in HAN by age group and sex.

In both ethnicities, the incidence rates of hypertension increased with age; in the same age group, men had higher incidence rate than that in the women.(**Figure 1** **and** **Figure 2**).

The adjusted relative risks of developing hypertension associated with each risk factor are presented by ethnicities in **Table 3** **and** **Table 4**. The incidence of hypertension was positively correlated with age, physical activity, drinking, BMI, family history of hypertension and prehypertension status in the Han population. In the Mongolian population, hypertension was positively correlated with age, physical activity, education level, drinking, BMI, prehypertension status and family history of hypertension.

**Table 3:** Adjusted hazards ratio for hypertension in HAN of rural Chinese.

Predictors	MAN (n = 10326)				WOMEN (n = 9921)			
	Univariate analysis		Multivariate analysis*		Univariate analysis		Multivariate analysis*	
	HR(95%CI)	P value	HR(95%CI)	P value	HR(95%CI)	P value	HR(95%CI)	P value
Age,yrs (1 SD increased)	1.028(1.025–1.030)	<0.001	1.033(1.029–1.036)	<0.001	1.033(1.030–1.037)	<0.001	1.035(1.032–1.038)	<0.001
High school education (yes vs no)	1.117(0.980–1.275)	0.098	-	-	0.953(0.783–1.153)	0.618	-	-
Family history of hypertension (yes vs no)	0.945(0.853–1.046)	0.276	-	-	1.143(1.007–1.297)	0.039	1.305(1.146–1.486)	<0.001
Drinking (yes vs no)	1.060(0.994–1.131)	0.077	1.116(1.044–1.192)	0.001	1.166(0.986–1.378)	0.073	-	-
Smoking (yes vs no)	0.949(0.887–1.015)	0.128	-	-	1.184(1.075–1.305)	0.001	-	-
Prehypertension (yes vs no)	1.200(1.109–1.300)	<0.001	1.187(1.095–1.287)	<0.001	1.353(1.251–1.463)	<0.001	1.210(1.117–1.311)	<0.001
BMI, kg/m^2^ (1 SD increased)	1.020(1.008–1.032)	0.001	1.030(1.018–1.040)	<0.001	1.035(1.024–1.046)	0.001	1.043(1.032–1.054)	<0.001
Physical activity								
moderate	1.000(reference)		1.000(reference)		1.000(reference)			
low	1.143(1.049–1.246)	0.002	0.879(0.801–0.964)	0.006	1.414(1.299–1.539)	<0.001	-	-
high	1.086(1.008–1.170)	0.029	1.143(1.061–1.232)	<0.001	0.937(0.854–1.029)	0.172	-	-

HR: hazards ratio; 95%CI: 95% confidence interval;

* adjusted for age, high school education, family history of hypertension, drinking, smoking, prehypertension, BMI, physical activity.

**Table 4:** Adjusted hazards ratio for hypertension in Mongolian of rural Chinese.

Variables	MAN(n = 2298)				WOMEN(n = 2455)			
	Univariate analysis		Multivariate analysis*		Univariate analysis		Multivariate analysis*	
	HR(95%CI)	P value	HR(95%CI)	P value	HR(95%CI)	P value	HR(95%CI)	P value
Age, yrs(1 SD increased)	1.0283(1.017–1.028)	<0.001	1.028(1.022–1.034)	<0.001	1.032(1.026–1.037)	<0.001	1.032(1.025–1.038)	<0.001
High school education(yes vs no)	1.201(0.990–0.457)	0.064	1.276(1.049–1.553)	0.015	0.971(0.777–1.213)	0.795	-	-
Family history of hypertension(yes vs no)	1.166(0.984–1.382)	0.077	1.297(1.090–1.544)	0.003	1.324(1.081–1.621)	0.007	1.615(1.314–1.986)	<0.001
Drinking (yes vs no)	1.113(0.982–1.261)	0.095	1.170(1.030–1.330)	0.016	0.974(0.765–1.240)	0.83	-	-
Smoking (yes vs no)	0.983(0.864–1.117)	0.789	-	-	1.075(0.900–1.283)	0.428	-	-
Prehypertension(yes vs no)	1.120(0.959–1.308)	0.152	-	-	1.556(1.346–1.798)	<0.001	1.433(1.235–1.663)	<0.001
BMI, kg/m^2^(1 SD increased)	1.009(0.987–1.031)	0.436	-	-	1.021(1.001–1.041)	0.042	1.021(1.002–1.041)	0.031
Physical activity								
moderate	11.000(reference)		1.000(reference)		1.000(reference)		1.000(reference)	
low	1.344(1.122–1.611)	0.001	1.011(0.831–1.229)	0.912	1.770(1.516–2.066)	<0.001	1.231(1.036–1.464)	0.018
high	1.322(1.160–1.606)	<0.001	1.383(1.212–1.577)	<0.001	1.204(1.077–1.399)	0.015	1.261(1.084–1.466)	0.003

HR: hazards ratio; 95%CI: 95% confidence interval;

* adjusted for age, high school education, family history of hypertension, drinking, prehypertension, BMI, physical activity.

**Table 5** presents the rates of hypertension awareness, treatment, and control by sex and age group. Overall, 42.3% Mongolian people with hypertension and 36.5% Han people with hypertension were aware of their diagnosis, only 18.2% of the Mongolians and 13.1% of the Hans were taking prescribed medication to lower their blood pressure, and only 1.3% of the Mongolians and 0.7% of the Hans achieved blood pressure control.

**Table 5:** Percentage of persons with incident hypertension who are aware, treated, and controlled, between Mongolian and Han.

Han(n = 6689)							Mongolian(n = 2090)					
	Aware, %		Treated, %		Controlled, %		Aware, %		Treated, %		Controlled, %	
	man	women	man	women	man	women	man	women	man	women	man	women
35–44	28.5	35.4	8.8	12.6	0.4	0.8	32.2	37.2	14.2	13.4	0.9	2.0
45–54	36.5	41.5	12.4	12.6	0.7	0.8	39.3	47.9	15.2	17.4	1.1	1.9
55–64	35.1	44.0	12.9	18.1	0.6	1.2	48.5	54.5	24.0	26.0	0.5	1.5
≥65	39.9	38.6	20.0	16.4	0.7	0.8	47.3	52.4	20.5	31.7	0.0	1.6
Total	33.9	39.7	12.2	14.3	0.6	0.9	39.1	45.9	17.0	19.5	0.8	1.8

## Discussion

We observed a high incidence rate of hypertension in both Mongolian and Han populations in northeast China. The majority of new cases were untreated and uncontrolled. We also identified a number of modifiable risk factors for hypertension in an environment of increasing urbanization and Westernization of diet and health behaviors in rural China.

Two studies conducted in China [2], [10] between 1980 and 2000 reported the incidence of hypertension to be between 2.3% and 5.2%.One study conducted in India between 2003 and 2010 reported the incidence of hypertension to be 3.3% [11], and a study conducted in Korean between 2003 and 2008 reported the incidence of hypertension to be 5.3% [12]. Two Western studies, one conducted in Canada [13] and the other conducted in the United States [14] reported annual hypertension incidences in those countries between 2.6% and 9.3%. The highest annual incidence of 9.3% occurred in study subjects who were previously classified as having prehypertension. Our study conducted in rural Liaoning province found an annual hypertension incidence of 9.77% in a Han population and 12.64% in a Mongolian population. Among subjects in populations who had been previously classified as having prehypertension, the annual hypertension incidence was 10.36% for Hans and 13.24% for Mongolians, which were higher than incidences reported by any of the other studies. Therefore, we conclude that the incidence of hypertension among rural Chinese adults accelerated during the past two decades. Now, the rate of hypertension in rural populations exceeds the incidence in urban Chinese and in most Asian and Western counties.

Hypertension is one of the most important risk factors for cardiovascular disease. It has been speculated that the risk factors for hypertension and incidence of hypertension in China may vary among different ethnic groups and regions of the country. We found that the incidence of hypertension in both Mongolian and Han populations is very high (12.64% and 9.77%, respectively). The mean BMI, and the prevalence of lipid disorders and diabetes in the populations we investigated were not as high as those observed in American and other Western countries, but the changes in lifestyle and diet such as increased drinking and smoking and high salt intake, as well as an increase in life expectancy, following economic development changes in China may explain the high incidence of hypertension. Additionally, a variety of genetic factors may also play a role in the development of hypertension.

Similar to the findings of other studies [15]–[17], the present study indicates that age, physical activity, drinking, BMI, family of hypertension and prehypertension are clearly associated with hypertension both in Mongolian and Han populations.

In the present study, prehypertension was independent predictor for incident hypertension, as has been reported previously [14], [18], [19]. One potential reason for the high incidence of hypertension among prehypertensive individuals is that risk factors for hypertension (such as higher baseline age, higher body mass index, and higher total cholesterol levels) were more common among prehypertensive individuals [20]. These findings support the recommendations of the JNC-7 report regarding prehypertensive individuals, lifestyle modification and multiple risk factor reduction.

The rates of awareness, treatment and control for incident hypertension in the present study were lower than those previously reported in urban China and other Asian countries [4], [11]. The reasons for the poor rates of awareness, treatment and control of hypertension in Mongolian populations and Han populations were likely multifactor, and may include poor health education, difficult access to medical care, and a lower income in rural areas of China. In the future, it will be important to increase public awareness of hypertension through the use of education programs and innovative health-care strategies.

There are several limitations to this study. First, follow-up BP measurements were taken only one time during the follow-up period, and the white-coat effect or natural variability in blood pressure may contributed to the “high” incidence rate of hypertension in the current study. Future studies should include repeated blood pressure measurements. Second, biochemical data related to hypertension was not collected in the current study and should be collected and analyzed in future studies.

The current study reported a high incidence rate of hypertension in the Mongolian and Han populations of rural China, and the vast majority of incident hypertension cases were untreated. Our results underscore the urgent need for developing a blood pressure education program to coordinate the efforts for detection, prevention, and treatment of hypertension in the rural areas of northeast China.
